# Preoperative inhalation therapy for patients with chronic obstructive pulmonary disease undergoing lung surgery: a retrospective study

**DOI:** 10.1186/s13019-022-02042-y

**Published:** 2022-11-24

**Authors:** Ryusuke Machino, Koichiro Shimoyama, Takeshi Nagayasu, Tsutomu Tagawa

**Affiliations:** 1 Chest Surgery, National Hospital Organization Nagasaki Medical Center, 2-1001-1 Kubaru, Omura, Nagasaki 856-8562 Japan; 2 Department of Surgical Oncology, Nagasaki University Graduate School of Biological Sciences, 1-7-1 Sakamoto, Nagasaki, 852-8501 Japan

**Keywords:** Lung surgery, Inhalation, Chronic obstructive pulmonary disease

## Abstract

**Background:**

Research shows that even the short-term administration of inhaled drugs immediately before surgery can improve respiratory function in surgical candidates with chronic obstructive pulmonary disease (COPD). However, the long-term efficacies of different types of long-acting inhaled agents when used during a short preoperative period remain unclear. Therefore, we evaluated the efficacies of short-term, preoperative long-acting muscarinic antagonists (LAMAs), inhaled corticosteroids with long-acting β2-agonists (ICSs/LABAs), and long-acting muscarinic antagonists with long-acting β2-agonists (LAMAs/LABAs) in patients with COPD after lung resection.

**Methods:**

Patients who underwent anatomical lung resections between April 2010 and March 2020 were divided into the non-COPD (193 patients) and COPD (241 patients) groups. The COPD group underwent preoperative treatment with either a LAMA (51 patients), an ICS/LABA (112 patients), or a LAMA/LABA (78 patients) for almost 1 month, with pulmonary function tests performed initially, just before surgery, and at 1 and 6 months after surgery. Improvement in preoperative respiratory function by inhalation therapy and the maintenance of improvement in respiratory function after surgery were examined in each group.

**Results:**

The COPD group had significantly higher proportions of men, older patients, smokers, and histopathologic types except for adenocarcinoma than the non-COPD group; however, there were neither differences in sex, age, percentage of smokers, or histopathologic type among the inhalant groups within the COPD group nor were there differences in percentage of GOLD stage, preoperative inhalation period, or percentage of resected lobes in lobectomy. Preoperative increases in forced expiratory volume in 1.0 s (FEV1.0) were significantly higher in the COPD group (129.07 ± 11.29 mL) than in the non-COPD group (-2.32 ± 12.93 mL) (*p* < 0.0001). At 6 months, there was no significant difference in residual FEV1.0 between the COPD-LAMA/LABA (2017.46 ± 62.43 mL) and non-COPD groups (2046.93 ± 40.53 mL). The FEV1.0 reduction rate was more suppressed in the COPD-LAMA/LABA group than in the non-COPD group at 1 and 6 months after surgery.

**Conclusions:**

Short-term, preoperative, inhaled pharmacotherapies, particularly LAMAs/LABAs, were effective at improving respiratory function in patients with COPD; thus, these agents are recommended for use in this population.

## Background

The number of patients with chronic obstructive pulmonary disease (COPD) who require surgical treatments is increasing as the population ages; however, even when patients with COPD are deemed healthy enough to undergo surgical procedures, they can sometimes experience postoperative respiratory depression and associated complications. Many clinical studies have shown an increased risk of perioperative death (30–50%) in patients with a predicted postoperative forced expiratory volume in 1.0 s (ppo-FEV1.0) of 40% or 1 L (ppo-FEV1.0 = preoperative FEV1.0 × [remaining lung segments/total lung segments]) [1–4].

In Japan, COPD treatment guidelines recommend regular use of long-acting bronchodilators in patients with Global Initiative for Chronic Obstructive Lung Disease (GOLD) stage II disease or higher [5]. Long-acting bronchodilators, including long-acting muscarinic antagonists (LAMAs) and long-acting β2-agonists (LABAs), are often used in combination with inhaled corticosteroids (ICSs) for the management of these patients, with many studies confirming the efficacy of this therapeutic approach for COPD [6–11].

Research has shown that the administration of tiotropium (a LAMA) during the 2 weeks preceding surgery can significantly improve respiratory symptoms and lung function, as well as reduce the frequency of postoperative complications [12]. Furthermore, the SHINE study showed that the administration of LAMAs/LABAs improved respiratory function in patients with COPD after only 2 weeks of use. It has also been reported that the postoperative FEV1.0 and diffusing capacity for carbon monoxide (DLco) are factors that determine not only the risk of postoperative complications, but also patient prognosis [13, 14]. It is therefore likely that inhaled drugs can improve respiratory function in surgical candidates with COPD, even when administered over a relatively short period immediately preceding surgery [11].

However, to the best of our knowledge, there have been no previous studies comparing the long-term efficacies of these three types of long-acting inhaled agents when used during a short preoperative period. In our department, all patients who have undergone lung resections participate in pulmonary rehabilitation from the initial preoperative examination until immediately before surgery. In addition, all patients with COPD are preoperatively treated with either LAMAs, ICSs/LABAs, or LAMAs/LABAs, depending on the drug released at that time. Bias was avoided as the inhaler was not specifically selected for each patient. Therefore, this study aimed to compare the efficacy of preoperative respiratory rehabilitation with and without these three types of inhalation therapies in patients who have undergone lung surgeries.

## Methods

### Study design and study population

For this retrospective study, we searched the electronic medical record database of our institution to identify 848 patients who had undergone anatomical lung resections regardless of their underlying disease, including lung cancer or metastatic lung tumors, between April 2010 and March 2019 at our hospital. While neither patient age nor preexisting conditions were considered as exclusion criteria, patients who underwent partial resections were not included in this study because their postoperative decreases in lung function were thought to be less substantial than those of patients undergoing anatomical lung resections and because patients who originally had poor lung function and chose to undergo reduction surgery were included. We excluded 414 patients (1) who appear to have low pulmonary function due to narrowing or obstruction of the central airway by the tumor (2) who had undergone previous inhalation therapy for treatment of COPD, asthma, or other respiratory conditions at the time of their initial preoperative examination; (3) whose postoperative follow-up was conducted at another hospital; or (4) whose postoperative respiratory function could not be assessed. Thus, 434 patients were included in this study. The patients placed in the COPD group were potentially unaware of their condition.

All patients in this study underwent respiratory function testing at their initial medical examination. Using these results, patients were classified into either the COPD group (FEV1.0% ≤ 70%) (241 patients) or the non-COPD group (FEV1.0% > 70%) (193 patients). At this time, the severity of COPD was also evaluated based on the GOLD criteria [15].

### Study interventions

Regardless of study group, all patients who smoked were counseled regarding smoking cessation beginning at their initial medical examination. In addition, all study patients received preoperative respiratory rehabilitation with an incentive spirometer (COACH 2™; Smiths Medical, Minneapolis, MN, USA). Patients in the COPD group also initiated inhalation therapy with either a LAMA (COPD-LAMA group; 51 patients; April 2010 to July 2012), an ICS/LABA (COPD-ICS/LABA group; 112 patients; August 2012 to January 2016), or a LAMA/LABA (COPD-LAMA/LABA group; 78 patients; February 2016 to March 2020), with modifications in pharmacotherapy regimens as drugs became available. The choice of drug was determined only by the launch date of the drug, and only one drug was used during a specific period. The drug was prescribed according to the results of respiratory function testing at the initial examination and was used until admission for surgery. The dosage and administration volume of the inhalant used in this study were as per manufacturer’s instructions. Therapeutic interventions were stopped just before surgery, and no study-related postoperative therapeutic interventions were performed owing to poor medication compliance because the majority of patients had mild COPD and lacked subjective symptoms.

### Patient evaluations and comparisons

**Fig. 1:**
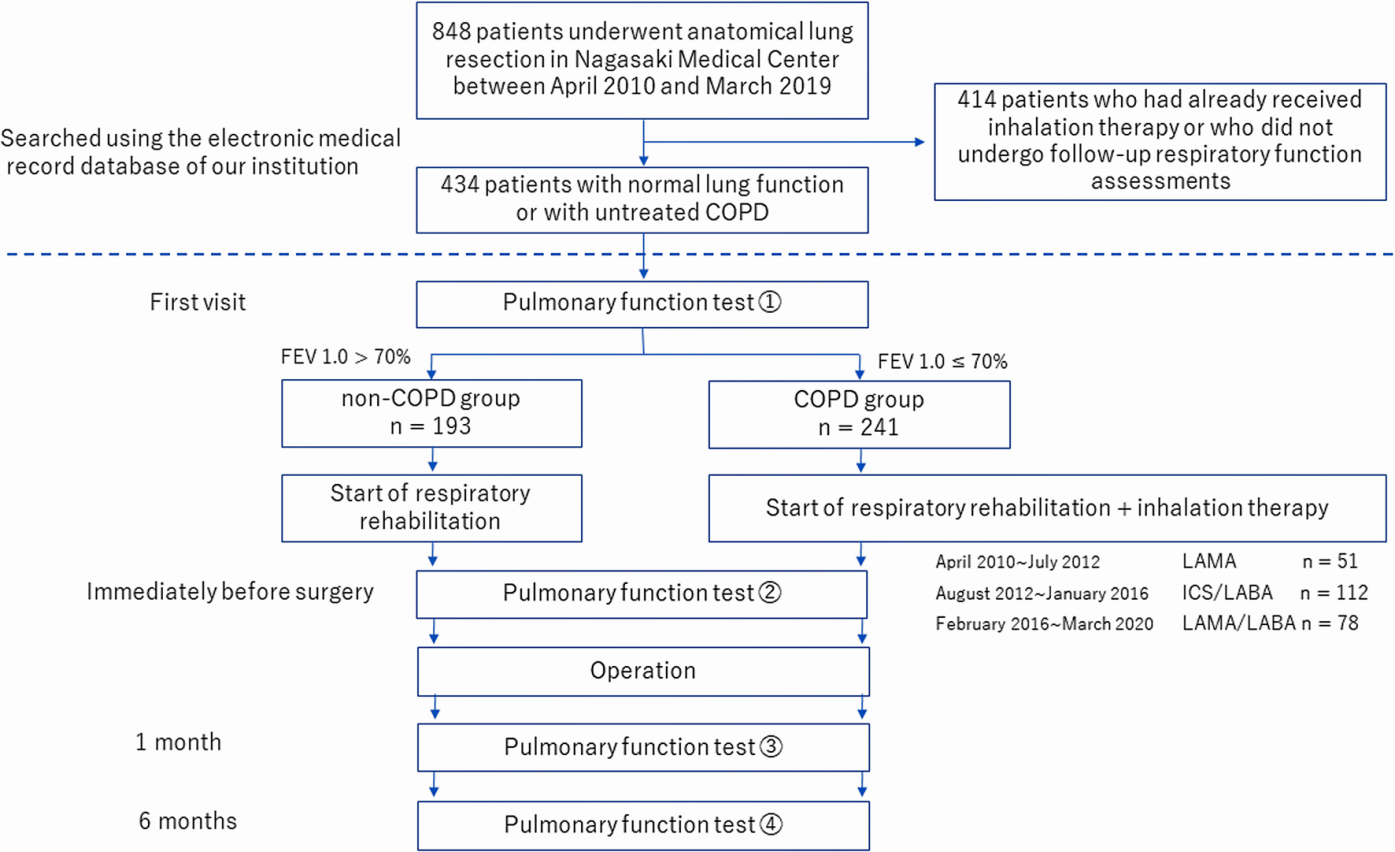
Study flowchart showing the clinical protocol for performing respiratory function testing of study patients. COPD, chronic obstructive pulmonary disease; FEV1.0, forced expiratory volume in 1.0 s; ICS, inhaled corticosteroid; LABA, long-acting β2-agonist; LAMA, long-acting muscarinic antagonist

Additional respiratory function testing was performed immediately before surgery, at 1 month after surgery, and at 6 months after surgery. A flowchart of the protocol used for respiratory function testing and treatment in study patients is presented in Fig. 1.

Patient age; sex; history of hypertension, diabetes, and/or smoking; duration of the preoperative intervention; vital capacity (VC) and %VC; FEV1.0, %FEV1.0, and FEV1.0%; %DLco at the initial visit, pathological diagnosis (adenocarcinoma, squamous cell carcinoma, and other pathologic type) were compared between the four study groups (non-COPD, COPD-LAMA, COPD-ICS/LABA, and COPD-LAMA/LABA). Initial GOLD stages were also compared between the three COPD subgroups (COPD-LAMA, COPD-ICS/LABA, and COPD-LAMA/LABA).

The amount of change in FEV1.0 (i.e., FEV1.0 after intervention—FEV1.0 before intervention), amount of change in %FEV1.0 (i.e., %FEV1.0 after intervention—%FEV1.0 before intervention), and rate of change in FEV1.0 (i.e., FEV1.0 after intervention/FEV1.0 before intervention) in each group were also compared based on data from the initial visit, preoperative visit, and 1- and 6-month postoperative visits.

For patients undergoing lobectomies (with the exception of those undergoing segmentectomies, combined resections with surrounding organs, bi-lobectomies, bronchoplasties, or pneumonectomies), the amount of change in FEV1.0 and rate of change in FEV1.0 of each group immediately before surgery and at 1 and 6 months after surgery were compared with the FEV1.0 at the initial visit. Additionally, the rate of change in the FEV1.0 at 1 and 6 months after surgery was compared with the FEV1.0 values obtained immediately before surgery in all study groups.

### Statistical analyses

Data are presented as the mean ± standard deviation or the median with interquartile range. Comparisons between two groups were assessed using Student’s *t*-tests for normally distributed variables. Comparisons between three or four groups were assessed using analysis of variance, with comparisons between each group assessed by the Tukey–Kramer method. A *p*-value of < 0.05 was considered reflective of statistical significance. The JMP software program, version 11 (SAS Institute Inc., Cary, NC, USA), was used for all statistical analyses.

## Results

### Patient characteristics

The clinical characteristics of patients in the non-COPD (193 patients) and COPD groups (241 patients) are shown in Table 1. The COPD group was found to be significantly older with higher proportions of men and smokers than the non-COPD group. The initial VC was also significantly higher in the COPD group, while the initial FEV1.0, %FEV1.0, FEV1.0%, and %DLco were significantly lower in the COPD group. There were no significant differences in the lengths of preoperative intervention periods or the types of surgical procedures between these two groups. The COPD group had significantly more patients except for adenocarcinoma than the non-COPD group.

**Table 1:** Characteristics of patients in the non-COPD and COPD groups

Characteristics		Non-COPD Group (n = 193)	COPD Group (n = 241)	*p* value
Sex	(male/female)	85/108	198/43	< 0.0001
Age	(years)	67.81 ± 0.58	71.51 ± 0.52	< 0.0001
Hypertension	( +)/(–)	91/102	136/105	0.066
Diabetes mellitus	( +)/(–)	40/153	49/192	1.00
Smoking	( +)/(–)	80/113	205/36	< 0.0001
Intervention Period	(days)	23.65 ± 0.93	23.47 ± 0.83	0.886
VC	(mL)	2993.68 ± 52.69	3272.61 ± 47.16	< 0.0001
%VC	(%)	109.30 ± 1.24	107.59 ± 1.11	0.308
FEV1.0	(mL)	2325.60 ± 37.44	2016.43 ± 33.50	< 0.0001
%FEV1.0	(%)	117.04 ± 1.54	94.31 ± 1.38	< 0.0001
FEV1.0%	(%)	78.63 ± 0.49	62.26 ± 0.44	< 0.0001
%DLco	(%)	119.65 ± 2.17	106.33 ± 1.91	< 0.0001
Pathology	Ad	157	138	< 0.001
	Sq	21	66	
	Other	8	37	
*Surgical procedure; Lobectomies*				
LUL		34	48	0.485
LLL		32	34	
RUL		53	77	
RLL		36	48	
RML		16	9	
*Surgical procedure; Reduction surgery and enlargement surgery*				
Segmentectomy		19	22	
Extended Surgery		3	4	

Values are shown as numbers or mean ± standard deviation. *P* < 0.05 indicates statistical significance

Characteristics of the three COPD subgroups are shown in Table 2. The mean initial VC and %VC were significantly higher in the COPD-LAMA/LABA subgroup than in the COPD-ICS/LABA subgroup. The mean initial %DLco was significantly higher in the COPD-LAMA/LABA subgroup than in the COPD-LAMA subgroup. There were no significant differences in the resected lobes between the three subgroups of patients with COPD who had undergone lobectomy with long-term respiratory function follow-up, except for in the frequencies of segmentectomies and extended surgeries (bi-lobectomies, bronchoplasties, or combined resections with surrounding organs). There were no significant differences in any other patient characteristics, including the duration of the preoperative intervention period, eosinophil count in peripheral blood, maximum inspiratory flow-volume curve. There were more patients with GOLD II and III COPD in the squamous cell carcinoma group and the other group than in the adenocarcinoma group, with a significant difference, in COPD-ICS/LABA group (Table 3).

**Table 2:** Characteristics of patients in the COPD subgroups

Characteristic		LAMA group (n = 51)	ICS/LABA group (n = 112)	LAMA/LABA group (n = 78)	*p* value
Sex	(male/female)	42/9	91/21	65/13	0.933
Age	(years)	71.98 ± 1.13	71.93 ± 0.77	70.62 ± 0.92	0.426
Hypertension	( +)/(–)	28/23	68/44	40/38	0.422
Diabetes mellitus	( +)/(–)	10/41	23/89	16/62	0.989
Smoking	( +)/(–)	45/6	93/19	67/11	0.667
Intervention period	(days)	25.88 ± 1.81	22.52 ± 1.22	23.26 ± 1.46	0.348
VC	(mL)	3184.71 ± 102.60	**3162.32 ± 69.23**	**3488.46 ± 82.96**	0.0079(ICS/LABA vs. LAMA/LABA)
%VC	(%)	104.53 ± 2.63	**105.70 ± 1.77**	**112.32 ± 2.12**	0.0462(ICS/LABA vs. LAMA/LABA)
FEV1.0	(mL)	1975.29 ± 69.05	1963.84 ± 46.59	2118.85 ± 55.83	0.0845
%FEV1.0	(%)	95.84 ± 3.10	93.24 ± 2.07	94.86 ± 2.48	0.757
FEV1.0%	(%)	64.01 ± 1.10	62.25 ± 0.74	61.13 ± 0.89	0.125
%DLco	(%)	**97.74 ± 4.18**	105.43 ± 2.74	**113.23 ± 3.36**	0.02(LAMA vs. LAMA/LABA)
*GOLD stage*					
I		41	80	63	0.816
II		10	30	14	
III		0	2	1	
Pathology	Ad	24	64	50	0.094
	Sq	14	30	22	
	Other	13	18	6	
*Surgical procedure; Lobectomies*					
LUL		12	18	18	0.708
LLL		7	13	14	
RUL		16	38	23	
RLL		12	19	16	
RML		0	5	4	
*Surgical procedure; Reduction surgery and enlargement surgery*					
Segmentectomy		2	19	1	0.001
Extended Surgery		2	0	2	

Data are presented as numbers or the mean ± standard deviation. *P* < 0.05 indicates statistical significance. Bold text indicates statistical significance

**Table 3:** GOLD stage by pathologic type in the COPD subgroups

GOLD stage	Pathology				*p* value
	Ad	Sq	Other	Total	
*LAMA group (n* = *51)*					
I	19	10	11	40	0.913
II	4	4	2	10	
III	1	0	0	1	
Total	24	14	13	51	
*ICS/LABA group (n* = *112)*					
I	50	20	12	82	**0.017**
II	14	10	4	28	
III	0	0	2	2	
Total	64	30	18	112	
*LAMA/LABA group (n* = *78)*					
I	40	17	6	63	0.694
II	9	5	0	14	
III	1	0	0	1	
Total	50	22	6	78	

Data are presented as numbers. *P* < 0.05 indicates statistical significance. Bold text indicates statistical significance

### Efficacy of bronchodilators before surgery

**Fig. 2:**
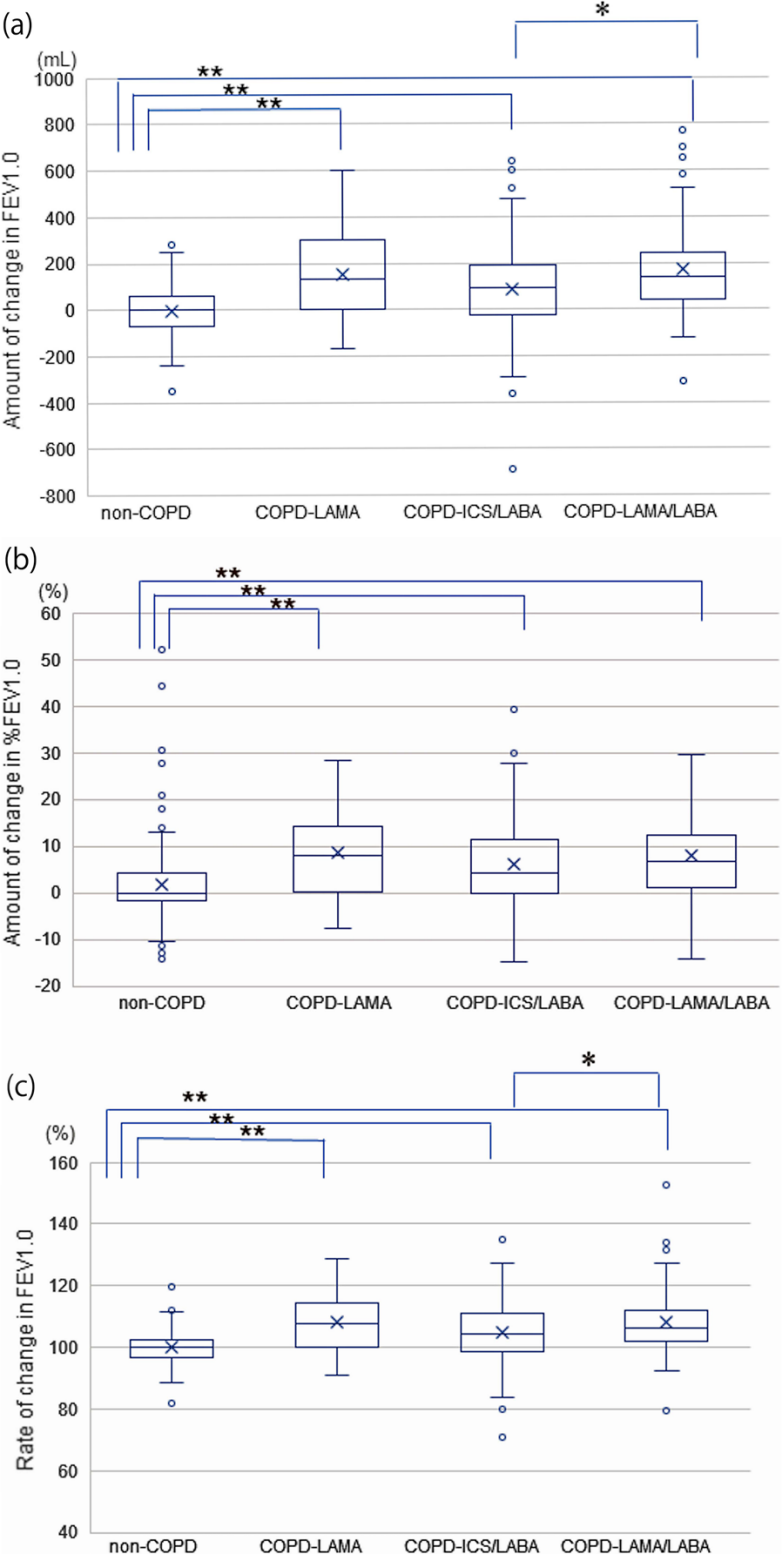
Preoperative changes in FEV1.0 and %FEV1.0 after study interventions in the four study groups. **a** Amount of change in FEV1.0; **b** amount of change in %FEV1.0; and **c** rate of change in FEV1.0. *p < 0.05, **p < 0.01. COPD, chronic obstructive pulmonary disease; FEV1.0, forced expiratory volume in 1.0 s; ICS, inhaled corticosteroid; LABA, long-acting β2-agonist; LAMA, long-acting muscarinic antagonist

The amount and rate of change in FEV1.0 and the amount of change in %FEV1.0 after preoperative interventions in the four study groups are shown in Fig. 2. In the non-COPD group, there was almost no change in the mean FEV1.0 between the initial and preoperative visits (mean amount of change: -2.32 ± 12.76 mL [mean rate of change: 0.12 ± 0.71%]); however, the COPD-LAMA group showed a significant improvement in the FEV1.0 of 153.40 ± 23.83 mL (8.42 ± 1.35%), the COPD-ICS/LABA group showed an improvement of 87.35 ± 16.50 mL (4.19 ± 0.95%), and the COPD-LAMA/LABA group showed an improvement of 171.14 ± 19.53 mL (8.04 ± 1.05%), all of which were significantly improved in comparison with the non-COPD group (Fig. 2a). The amounts of change in %FEV1.0 were significantly higher in the three COPD subgroups than in the non-COPD group; however, no significant differences were identified in the amounts of change in %FEV1.0 between the three COPD subgroups (Fig. 2b). The amounts and rates of change in FEV1.0 were significantly higher in the COPD-LAMA/LABA group than in the COPD-ICS/LABA group (Fig. 2c). No significant differences in amount and rate of improvement in FEV1.0 by pathologic type in the two COPD subgroups (COPD-LAMA group: adenocarcinoma; 163.33 ± 42.56 mL [8.47 ± 2.08%], squamous cell carcinoma; 145.00 ± 38.04 mL [8.75 ± 3.04%], other; 145.83 ± 53.43 mL [7.83 ± 2.13%],, COPD-LAMA/LABA group: adenocarcinoma; 158.94 ± 26.51 mL [7.59 ± 1.36%], squamous cell carcinoma; 207.06 ± 44.51 mL [10.91 ± 3.17%], other; 165.00 ± 127.35 mL [7.95 ± 5.68%]). However, in the COPD-ICS/LABA group, both the amount (*p* = 0.037) and rate (*p* = 0.040) of improvement in FEV1.0 of squamous cell carcinoma and other were significantly higher than that of adenocarcinoma (COPD-ICS/LABA group: adenocarcinoma; 49.64 ± 25.69 mL [2.98 ± 1.39%], squamous cell carcinoma; 108.21 ± 40.27 mL [6.38 ± 2.39%], other; 186.67 ± 44.88 mL [9.26 ± 1.95%]).

### Efficacy of bronchodilators after surgery (examinations in lobectomy cases)

**Fig. 3:**
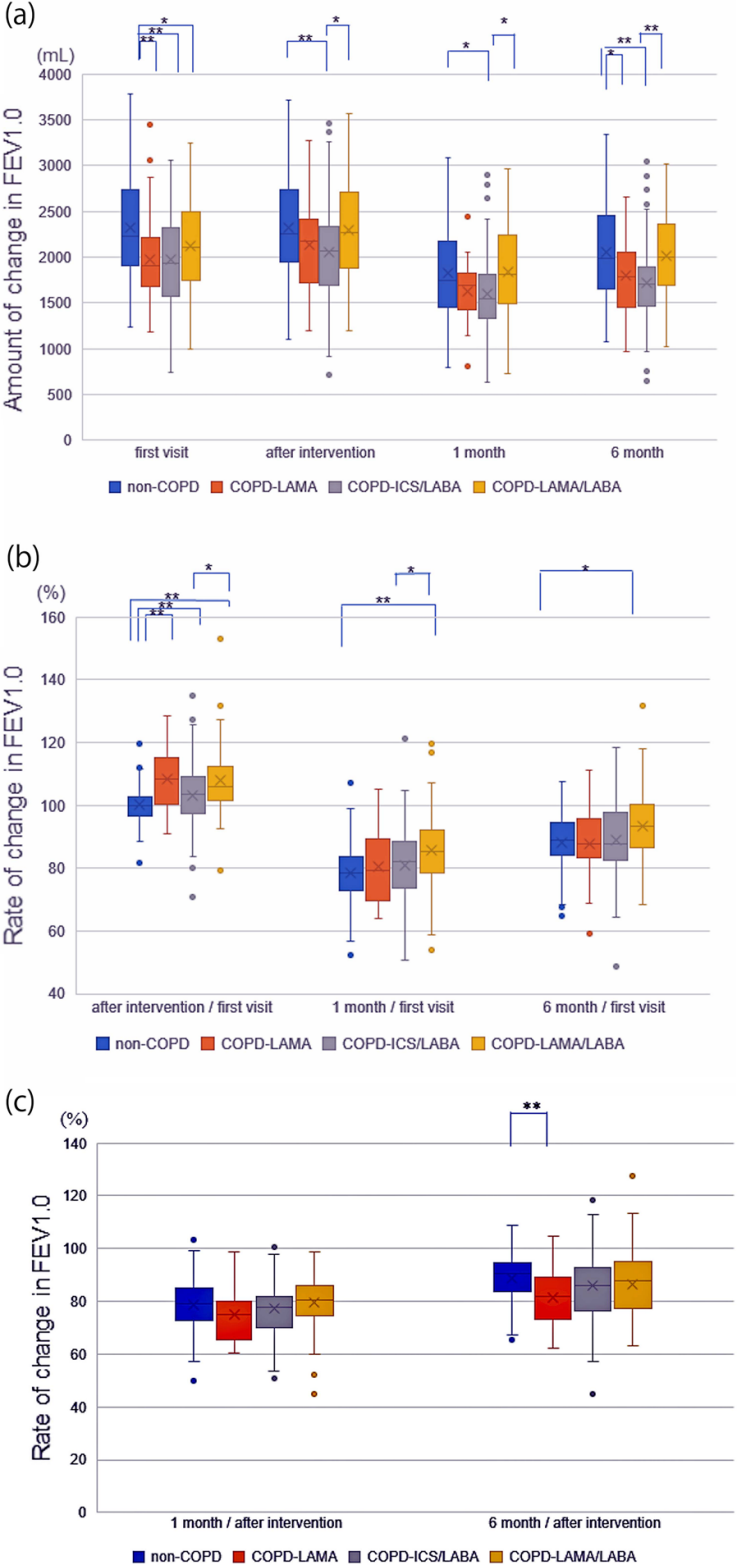
Comparison of FEV1.0 changes in the four groups at study time points. **a** Amount of change in FEV1.0 at study time points; **b** rate of change in FEV1.0 in comparison with the initial visit; and **c** rate of change in FEV1.0 at 1 and 6 months after surgery in comparison with immediately after preoperative interventions. *p < 0.05, **p < 0.01. COPD, chronic obstructive pulmonary disease; FEV1.0, forced expiratory volume in 1.0 s; ICS, inhaled corticosteroid; LABA, long-acting β2-agonist; LAMA, long-acting muscarinic antagonist

One month after surgery, there were no significant differences between the residual FEV1.0 of the non-COPD group (1826.30 ± 35.38 mL) and those of the COPD-LAMA (1630.80 ± 90.89 mL) and COPD-LAMA/LABA (1834.31 ± 53.56 mL) groups. Six months after surgery, there was also no significant difference between the residual FEV1.0 of the non-COPD group (2046.93 ± 40.53 mL) and that of the COPD-LAMA/LABA group (2017.46 ± 62.43 mL) (Fig. 3a). The rate of reduction in FEV1.0, however, was more significantly suppressed in the COPD-LAMA/LABA group than in the non-COPD group at 1 and 6 months after surgery (Fig. 3b). Comparison of FEV1.0 values obtained at 1 and 6 months after surgery to those obtained immediately before surgery demonstrated a significant difference in the rate of change in FEV1.0 between the COPD-LAMA and non-COPD groups at 6 months after surgery. There were, however, no other significant differences in the rates of change in FEV1.0 (Fig. 3c).

## Discussion

This study demonstrated that preoperative treatment with inhaled pharmacotherapies can be effective for the treatment of patients with COPD, even when used for only 1 month before surgery. Moreover, LAMAs/LABAs were found to be the most effective at improving perioperative FEV1.0 values and maintaining postoperative FEV1.0 values in these patients.

Makino et al. [16] reported that preoperative inhalation of LAMAs/LABAs increased FEV1.0 values more significantly than inhalation of LAMAs alone; however, in the present study, there were no significant differences in FEV1.0 increases between the LAMA and LAMA/LABA subgroups. This discrepancy may be explained by the different patient populations recruited for these two studies. While the study by Makino et al. targeted patients with moderate-to-severe COPD (> GOLD stage II), most of the patients with COPD in our study had GOLD stage I disease.

Conversely, patients with very low preoperative FEV1.0 and FEV1.0% have been reported to be less likely to lose ventilatory function after lobectomy [17], a finding that has been supported by other studies. Sekine et al. [18] demonstrated that the postoperative ventilatory function in patients with COPD who had undergone lower or middle-lower lobectomies was better preserved than predicted. This finding suggests that resections of emphysematous lung tissue may be beneficial for preserving lung function, even in patients with moderate COPD. It is possible that the lobectomy itself improved the respiratory function of patients with COPD in this study. However, as most of the patients in this study had GOLD stage I COPD and there were no significant differences in the proportions of patients with moderate or severe COPD between the three subgroups that received inhalation therapy, we believe that the differences in ventilatory function improvement in each group were largely due to the effects of the inhalants.

Although it has been shown that LABAs have a suppressive effect on COPD exacerbations, tiotropium (a LAMA) has been revealed to have a stronger suppressive effect on COPD exacerbations than salmeterol or indacaterol (both LABAs) [19, 20]. Therefore, combination therapy using both LAMAs and LABAs may be more effective than LAMAs alone for the treatment of patients with moderate-to-severe COPD. Conversely, LAMAs alone may be sufficient for preoperative use in patients with mild COPD. In addition, the present study demonstrated that LAMAs/LABAs improved FEV1.0 values more significantly than ICSs/LABAs, as has been reported in previous studies [21].

COPD is a risk factor for squamous histological subtypes in smokers [22], and in this study, the COPD group contained significantly more patients with squamous cell carcinoma than the non-COPD group, suggesting an association between COPD and squamous histological subtypes. On the other hand, in the COPD subgroup that underwent inhalation therapy, especially in the COPD-ICS/LABA group, preoperative inhalation improved FEV1.0 in the squamous cell carcinoma and other pathologic type groups more than in the adenocarcinoma group. It is possible that patients with severe COPD who were more likely to benefit from inhalation therapy were included in the squamous cell carcinoma and other groups within the COPD-ICS/LABA group, however, this may be a result of COPD being associated with cancers that contain squamous histological subtypes, such as squamous cell carcinoma.

According to Leo et al. [23], up to 50% of patients with COPD have aggravation of their disease after surgery, highlighting the importance of perioperative COPD management. Although some postoperative decline in lung function is inevitable, this study demonstrated that preoperative inhalation therapy suppressed respiratory functional declines during the 6 months following surgery. Moreover, there were no significant differences in the reduction rates of change in FEV1.0 at 1 and 6 months after surgery compared with the values obtained immediately preceding surgery in the four study groups. Therefore, if FEV1.0 values immediately preceding surgery are the same, it is likely that there will be no significant difference in FEV1.0 reduction rates between patients with and without COPD in the postoperative period. It is thus possible that the degree to which FEV1.0 is improved by inhalation therapy at the time of surgery is a key factor in suppressing postoperative decreases in FEV1.0. Of note, the LAMA/LABA group had the greatest suppression of the postoperative FEV1.0 reduction rate compared to the FEV1.0 at the initial visit. It is also feasible that decreased respiratory function deterioration after lung resections is effective at preventing COPD exacerbations and that preoperative inhalation therapies, especially LAMAs/LABAs, are effective at maintaining respiratory function after lung resections in patients with COPD. Since this study was conducted in patients with COPD without subjective symptoms, inhalation therapy was not continued after surgery; however, because these patients were not experiencing subjective symptoms, patient compliance was a key consideration. It may be possible to maintain a high FEV1.0 value by continuing inhalation therapy even after surgery.

This study had some limitations. Our data were exclusively collected at a single center, and the number of patients in each group varied due to the study’s retrospective design. This study did not compare values between patients with COPD who did or did not undergo preoperative interventions. Overcoming this limitation would require a control group comprising COPD patients who do not receive inhalation therapy or non-COPD patients who receive inhalation therapy. However, as the efficacy of inhalation therapy is known, not inducing inhalation among COPD patients presents ethical challenges, as does administering inhalation therapy to non-COPD patients. Additionally, since the clinical benefit of administering LAMAs/LABAs to patients with COPD has been shown, using a more rigorous, randomized-controlled design to confirm the findings of this study could also raise ethical concerns. Compliance with treatment in patients with COPD without subjective symptoms was only confirmed verbally, and there may have been patients who were prescribed an inhalation medication but did not take it. Although LAMAs/LABAs have been reported to reduce the risk of perioperative complications [16], we did not evaluate perioperative complication rates because patients who were followed up at other hospitals or did not have long-term postoperative follow-up were excluded from the study. In the future, it is desirable to conduct prospective long-term observational studies, including those with perioperative complications.

## Conclusions

This study demonstrated that preoperative use of inhaled pharmacotherapies is effective at maintaining respiratory function in patients with COPD who are undergoing lung resections, even when used for only a 1-month period before surgery. Of the three inhaled agents evaluated in this study, LAMAs/LABAs were found to be the most effective at improving patients’ perioperative FEV1.0 values and maintaining postoperative FEV1.0 values, and these agents are recommended for use in this population.

## Data Availability

The datasets used and/or analyzed during the current study are available from the corresponding author on reasonable request.

## Abbreviations

Ad: Adenocarcinoma
COPD: Chronic obstructive pulmonary disease
DLco: Diffusion capacity for carbon monoxide
FEV1.0: Forced expiratory volume in 1.0 s
GOLD: Global Initiative for Obstructive Lung Disease
ICS: Inhaled corticosteroid
LABA: Long-acting β2-agonist
LAMA: Long-acting muscarinic antagonist
LLL: Left lower lobectomy
LUL: Left upper lobectomy
ppo-FEV1.0: Predicted postoperative forced expiratory volume in 1.0 s
RLL: Right lower lobectomy
RML: Right middle lobectomy
RUL: Right upper lobectomy
Sq: Squamous cell carcinoma
VC: Vital capacity
